# Youth Impulsivity as a Moderator in the Relationship Between Parental Punitive Discipline and Child-to-Parent Violence

**DOI:** 10.3390/bs16060936

**Published:** 2026-06-06

**Authors:** M. Carmen Cano-Lozano, Lourdes Contreras, María J. Navas-Martínez

**Affiliations:** Department of Psychology, University of Jaén, 23071 Jaén, Spain; lmcontre@ujaen.es (L.C.); mjnavas@ujaen.es (M.J.N.-M.)

**Keywords:** child-to-parent violence, impulsivity, parental discipline, young adults

## Abstract

While recent studies have begun to investigate the interaction between individual characteristics and parenting practices in the development of child-to-parent violence, empirical evidence remains limited, particularly among young adults. This study examines the role of youth impulsivity as a moderator of the relationship between parental punitive discipline and child-to-parent violence. The sample consisted of 1041 young adults (51.1% women, 48.9% men), aged between 18 and 25 years (*M_age_* = 21.41, *SD* = 1.94), who lived with their parents. Participants completed the Child-to-Parent Violence Questionnaire, the Inventory of Parental Discipline Methods, and the Barratt Impulsivity Scale. Results indicated that parental punitive discipline and impulsivity were positively associated with violence toward both fathers and mothers. Impulsivity moderated only the relationship between punitive maternal discipline and child-to-parent violence (toward both the father and the mother). Specifically, this effect was stronger at higher levels of impulsivity. These findings highlight the importance of considering both individual traits and parental practices, as well as their interaction, in understanding child-to-parent violence during emerging adulthood. They also have important theoretical and practical implications for prevention and intervention.

## 1. Introduction

Violence perpetrated by sons and daughters against their parents, commonly referred as child-to-parent violence (hereinafter CPV), is the only type of family violence in which the aggressors are dependent on their victims, and remains one of the least studied forms in the literature (Rogers & Ashworth, 2024). This is the case even though this type of violence is currently recognized as a serious social problem with important negative consequences for the psychosocial health of the family as a whole (e.g., Beckmann et al., 2021; Cano-Lozano et al., 2022; Contreras et al., 2026).

Although there is no single consensus on the definition of CPV, a significant proportion of studies conceptualize this phenomenon as any act of physical, psychological, or financial violence (Cottrell, 2001) that is carried out consciously, intentionally, and repeatedly over time (Pereira et al., 2017) by a child toward a parent or a parental figure (Molla-Esparza & Aroca-Montolío, 2018), with the aim of controlling and dominating them (Cottrell, 2001; Howard & Rottem, 2008).

The most recent element incorporated into the definition of CPV is the habitual cohabitation between parents and children (Ibabe, 2020). This responds to a growing need to broaden the developmental period from which CPV is analyzed. Specifically, research on CPV has focused primarily on the adolescent stage (e.g., Bautista-Aranda et al., 2023; Jiménez-García et al., 2022; Margolin & Baucom, 2014; Navas-Martínez et al., 2025). However, particularly in Western countries, it is increasingly common for children to live with their parents beyond adolescence, that is, during emerging adulthood. This is the case in Spain, which ranks among the five European Union countries with the highest delay in the age at which children leave the family home (Eurostat, 2024). This developmental stage spans ages 18 to 25 (Arnett, 2000). It is also the focus of an emerging line of research which suggests that conflicts, tensions, and CPV may persist while parents and children live together in the family home (Burgos-Benavides et al., 2025; Cano-Lozano et al., 2021b; Ibabe et al., 2020; Johnson et al., 2022; Navas-Martínez et al., 2025; Simmons et al., 2020).

Although still scarce, studies that have analyzed the prevalence of CPV in community samples of young adults show high rates of this type of violence even at this developmental stage, underscoring the need for greater attention. Specifically, CPV ranges from 8.5% to 20% in an Australian sample (Simmons et al., 2020). When broken down by type, studies in Spanish (Ibabe et al., 2020), Italian (Navas-Martínez et al., 2025), and Ecuadorian samples (Burgos-Benavides et al., 2025) report psychological CPV ranges from 44% to 76%, physical CPV from 2% to 5.6%, financial CPV from 19.5% to 37%, and control and domain behaviors from 39% to 82%. These prevalence rates show considerable variability, which is also observed in adolescent samples. This variability is largely due to the lack of consensus in the conceptualization of CPV as well as in the criteria used to determine its presence.

One of the most frequently studied aspects of CPV is the analysis of risk factors. Junco-Guerrero et al. (2025), in a recent systematic review, identified several variables that predict the development of CPV, including individual, family, and social variables. In line with the nested ecological theory of CPV (Cottrell & Monk, 2004), the variables closest to the children—that is, family and individual factors—are those that exert the greatest influence on the development of CPV. However, significant gaps remain in our understanding of the family and individual variables associated with the development of CPV, particularly in young adult samples.

### 1.1. Family Characteristics: Parental Punitive Discipline as a Risk Factor

With regard to family variables, exposure to family violence is one of the most consistent predictors of CPV internationally (Gallego et al., 2019) in both adolescent (Bautista-Aranda et al., 2023; Contreras et al., 2020, 2026; Hernández et al., 2020; Navas-Martínez & Cano-Lozano, 2023) and young adult samples (Cano-Lozano et al., 2021b; Ibabe et al., 2020; Simmons et al., 2020).

Social learning theory has frequently been used as an explanatory framework for these findings (Bandura, 1977; Bandura & Walters, 1959). From this perspective, children from violent homes may learn, through observation and imitation of role models, that aggression is an appropriate strategy for resolving interpersonal conflicts.

In relation to direct exposure to parental aggression, there is a broad scientific consensus on the adverse effects of punitive disciplinary practices. Harsh parenting strategies have consistently been associated with both internalizing problems (e.g., anxiety and depression) and externalizing problems (e.g., disruptive and aggressive behaviors) in children and adolescents (MacKenzie et al., 2012; Pinquart, 2017).

In the context of CPV, most studies have found relationships between punitive disciplinary practices involving corporal punishment and/or psychological aggression and CPV (e.g., Beckmann et al., 2021; Cano-Lozano et al., 2022; Del Hoyo-Bilbao et al., 2018, 2020; Gámez-Guadix & Calvete, 2012; Pagani et al., 2009). However, findings regarding its predictive role are inconsistent. Some studies identify it as a predictor of CPV (Del Hoyo-Bilbao et al., 2018; Margolin & Baucom, 2014), and both toward father and mother (Cano-Lozano et al., 2022; Pagani et al., 2009), and others find that it predicts violence only toward fathers (Jiménez-Granado et al., 2023). While it is common in the field of CPV to develop separate models to predict CPV toward fathers and mothers, studies analyzing paternal and maternal punitive discipline separately are still scarce. This distinction could help clarify the role of this variable. Moreover, these discrepancies may be due to the involvement of additional variables. Specifically, other factors may mediate or moderate these relationships, suggesting that simple associations between these variables may be insufficient. In this regard, Del Hoyo-Bilbao et al. (2020) found that corporal punishment and psychological aggression predict CPV toward both fathers and mothers through other individual variables. This suggests that the impact of parental discipline may vary, among other factors, depending on the presence of individual vulnerability factors.

### 1.2. Individual Characteristics: Impulsivity as a Risk Factor

Regarding individual variables, several studies have identified distinguishing characteristics among adolescents who perpetrate CPV. In particular, it has been associated with a range of cognitive and emotional factors. Adolescents who engage in CPV tend to show a more hostile perception of parental authority, lower levels of self-esteem and empathy, and greater deficits in interpersonal conflict resolution skills compared to their non-CPV adolescents (Contreras & Cano-Lozano, 2015; Martín & Cortina, 2023). In addition, CPV has been linked to several cognitive processing patterns, including greater hostile attribution, rapid access to aggressive responses, anticipation of positive outcomes of aggression, and moral disengagement (Bautista-Aranda et al., 2023; Calvete et al., 2015; Contreras et al., 2020). The justification of violence has also been associated with CPV. Studies conducted with community samples have also found that the justification of violence predicts CPV (Contreras et al., 2020; Junco-Guerrero et al., 2021). Loinaz and Ma de Sousa (2020) observed that, in judicial samples, CPV offenders exhibit more justification of violence than in clinical samples.

With respect to emotional variables, studies conducted with adolescents indicate that anger is associated with CPV and is a significant predictor of this type of violence (Armstrong et al., 2018; Calvete et al., 2015; Cano-Lozano et al., 2020; Contreras et al., 2020). Similarly, research with young adults confirms that anger is a characteristic feature of individuals who perpetrate CPV and a significant predictor (Simmons et al., 2020).

Drug use has also been associated with CPV (Armstrong et al., 2018; Calvete et al., 2011) and has been identified as a predictor of this form of violence (Cano-Lozano et al., 2020; Del Hoyo-Bilbao et al., 2020).

Impulsivity has consistently been identified as a key variable in the development of CPV. It is conceptualized as difficulties in maintaining control over thoughts and behavior and in inhibiting responses (Barratt & Patton, 1983). A growing body of research has linked impulsivity to CPV (e.g., Calvete et al., 2011; Ibabe et al., 2007). Specifically, Contreras and Cano-Lozano (2015) found higher levels of impulsivity among adolescents who engage in CPV compared to those who do not. Similarly, a study with a community sample identified adolescent impulsivity as one of the main predictors of CPV (Del Hoyo-Bilbao et al., 2020).

Recent research has also examined impulsivity as a multidimensional construct. In particular, attentional and motor impulsivity have been identified as relevant dimensions in explaining CPV. However, the lack of planning dimension does not show a significant relationship with any type of CPV. The authors suggest that this may be because this type of violence is not usually planned (Rico et al., 2017). In samples of young adults, Simmons et al. (2020) found that participants who perpetrated mother abuse exhibited greater impulsivity in the context of emotional dysregulation than those who had not. In contrast, participants who perpetrated father abuse showed no significant differences compared to those who had not. In neither case did impulsivity show significant predictive power.

Although evidence supports the role of impulsivity in CPV, studies examining this relationship in young adults are scarce. Furthermore, it is important to know if the effect of impulsivity varies depending on whether the violence is directed toward the father or the mother.

### 1.3. Interaction Between Family and Individual Characteristics: Impulsivity as a Moderator

Beyond its direct relationship with violence, impulsivity may also function as an individual vulnerability factor that intensifies the effects of adverse family environments. This vulnerability may be partly explained by biased cognitive processing patterns. Reduced inhibitory control may lead adolescents to pay more attention to negative environmental elements and exhibit greater emotional reactivity in adverse contexts (Zhu et al., 2016). Consequently, they may be more likely to interpret parental discipline as hostile and respond with aggression.

From the perspective of the diathesis-stress model (Monroe & Simons, 1991), individuals who possess certain vulnerability characteristics -such as high impulsivity- may be more susceptible to environmental stressors, including parental harsh discipline. In this context, impulsivity may amplify the negative impact of punitive parenting practices, increasing the likelihood that adolescents respond with aggressive behavior.

Integrating this model with social learning theory, children with high impulsivity (diathesis, individual vulnerability) may tend to exhibit hostile attribution biases, leading them to interpret punitive discipline (stressor) as a personal attack or threat. This interpretation would more readily activate aggression as a learned behavior for resolving interpersonal conflicts. While social learning theory explains how violent behavior is acquired, the diathesis–stress model helps explain when and for whom such behavior is more likely to occur.

Empirical evidence supports the moderating role of impulsivity in various environmental stress contexts. Specifically, impulsivity has been found to intensify the relationship between peer victimization or parental rejection and the development of behavioral problems in adolescents (Y. Chen et al., 2021; Zhu et al., 2016). Similarly, the negative association between family warmth and delinquency appears to be significant only among adolescents with high levels of impulsivity (P. Chen & Jacobson, 2013). Other studies have reported that harsh discipline predicts externalizing behaviors among girls only at high levels of impulsivity (Leve et al., 2005).

Recent research has begun to explore the interaction between individual characteristics and parenting practices in CPV. Jiménez-Granado et al. (2023) found that inadequate parental discipline predicted CPV primarily among adolescents with elevated levels of psychopathic or borderline personality traits. Regarding the use of punitive discipline, Cano-Lozano et al. (2022), using a retrospective design with young adults aged 18 to 25 years, reported that parental stress, feelings of inefficacy, and impulsivity intensified the negative effects of punitive discipline on CPV. Only one study has examined the role of adolescent impulsivity in the relationship between parental harsh discipline and CPV (Gao et al., 2024), finding that the negative effects of parental harsh discipline on CPV were significantly stronger among adolescents with high levels of impulsivity, whereas this relationship was not significant among adolescents with lower levels of impulsivity.

In summary, the literature on the mechanisms that moderate the relationship between severe parental discipline and CPV toward fathers and mothers is still scarce, and, in particular, the role of impulsivity in this relationship in young adults.

### 1.4. The Current Study

The literature review has revealed several inconsistencies in the relationship between punitive discipline and CPV, and few studies have examined the differential role of paternal and maternal punitive discipline. Furthermore, there is limited evidence analyzing the role of impulsivity in CPV in young adults. Most studies of CPV have primarily focused on individual and family variables independently, whereas very few have examined the interaction between these factors. In addition, little empirical attention has been paid to the specific role of impulsivity in the relationship between parental punitive discipline and CPV. To the best of our knowledge, only Gao et al. (2024) have examined this mechanism of CPV in a sample of Chinese adolescents.

Given these limitations, several objectives arise. First, further evidence is needed on the role of paternal and maternal punitive discipline and youth impulsivity in CPV, as well as the interaction between these variables. Second, it is important to analyze these relationships in non-emancipated young adult samples to determine whether parental punitive discipline and impulsivity act as risk factors for CPV at this developmental stage, and whether impulsivity continues to amplify the negative impact of parental punitive discipline on CPV beyond adolescence. Third, it is necessary to develop separate models for CPV toward fathers and mothers, given previous evidence suggesting distinct mechanisms in each case.

For these reasons, this study aims to advance knowledge of CPV in a sample of Spanish young adults by examining the role of paternal and maternal punitive discipline, youth impulsivity, and their interaction in CPV toward father and mother. Specifically, three objectives were established.

1. To analyze the relationship and the predictive value of parental punitive discipline (father and mother) on CPV toward fathers and mothers. H1: Both paternal and maternal punitive discipline would be positively associated with and significant predictors of CPV toward both fathers and mothers. A more intense effect is expected within the same than across parental figures (Beckmann et al., 2021; Cano-Lozano et al., 2022; Del Hoyo-Bilbao et al., 2020).
2. To examine the association between youth impulsivity and CPV, toward both fathers and mothers, as well as its predictive value. H2: Youth impulsivity will be positively associated with and a significant predictor of CPV toward both fathers and mothers (Calvete et al., 2011; Contreras & Cano-Lozano, 2015; Ibabe et al., 2007).
3. To explore the moderating role of youth impulsivity in the relationship between paternal and maternal punitive discipline and CPV toward fathers and mothers. H3: Impulsivity will moderate this relationship, such that higher levels of impulsivity will strengthen the association between both paternal and maternal punitive discipline and CPV toward both fathers and mothers, with more intense effects within the same than between different parental figures (Gao et al., 2024).

## 2. Method

### 2.1. Participants

The sample comprised 1041 Spanish young adults (51.1% women and 48.9% men), aged between 18 and 25 years (*M_age_* = 21.41, *SD* = 1.94), who had lived with their parents during the past year and throughout adolescence (ages 12 and 17). Of these, 51.2% were university students and 48.8% were non-university students. Most participants (85.2%) were from Andalusia (southern Spain), with the remainder from the other 22 Spanish provinces. Regarding the family structure, 86.5% came from nuclear families, 8.3% from single-mother families, 1.6% from single-father families, and 3.7% from reconstituted families. In terms of monthly family income, the distribution was as follows: less than 1000 € (8.6%), between 1000–2000 € (48.5%), between 2000–3000 € (30.0%), and more than 3000 € (12.9%).

### 2.2. Instruments

Ad hoc Sociodemographic Questionnaire: It collects basic sociodemographic information on participants’ sex, age, nationality, place of residence, academic degree, type of family, and family income.

Child-to-Parent Violence Questionnaire, youth version (CPV-Q, Cano-Lozano et al., 2021a). It consists of 19 parallel items referring to different types of CPV: psychological, physical, and financial violence, and control/domain behaviors, toward the father (α = 0.841; α_o_ = 0.948; ω = 0.885; ω_o_ = 0.950) and the mother (α = 0.833; α_o_ = 0.946; ω = 0.879; ω_o_ = 0.949), separately. Participants indicated the frequency with which they had engaged in these behaviors over the last year (e.g., “I stole money from my parents”). Items are rated on a 5-point scale: 0 (never = never occurred), 1 (rarely = occurred once), 2 (sometimes = occurred two or three times), 3 (often = occurred four or five times), and 4 (very often = occurred six times or more). The CPV-Q has been used in multiple international studies, showing adequate psychometric properties, as well as good reliability and validity (Burgos-Benavides et al., 2025; Jiménez-García et al., 2020; Navas-Martínez et al., 2025).

Dimensions of Discipline Inventory (DDI-C, Straus & Fauchier, 2007; Spanish validation by Calvete et al., 2010). According to the authors of the instrument, punitive discipline (disciplinary method) is identified through the subscales of corporal punishment and psychological aggression, due to the high frequency of both behaviors and their shared function: the coercive control of the child. Parenting practices refer to the adolescent period (ages 12 to 17), allowing for a temporal separation between parenting practices and CPV. Each subscale includes 4 parallel items each (father: α = 0.859; α_o_ = 0.921; ω = 0.869; ω_o_ = 0.922; mother: α = 0.857; α_o_ = 0.917; ω = 0.862; ω_o_ = 0.917; e.g., “They gave you a push or forcefully pushed you aside”). Items were rated on a 5-point Likert-type scale: 0 = never, 1 = almost never, 2 = sometimes, 3 = usually, and 4 = always or almost always. This instrument is widely used internationally to evaluate disciplinary practices. Several studies with Spanish samples find adequate internal consistency, test–retest reliability, convergent and discriminant validity (Cano-Lozano et al., 2022; Del Hoyo-Bilbao et al., 2020; Gámez-Guadix et al., 2010; Ibabe et al., 2023).

Barratt Impulsivity Scale (BIS-11, Patton et al., 1995; Spanish validation by Oquendo et al., 2001). This scale evaluates the presence of a pattern of impulsivity behavior maintained over time. The degree of impulsivity was measured using attentional and motor impulsivity subscales (19 items: α = 0.696; α_o_ = 0.750; ω = 0.781; ω_o_ = 0.819) with a Likert response format: 1 = rarely/never, 2 = occasionally, 3 = often, and 4 = almost always/always (e.g., “I concentrate easily”). The dimension of “lack of planning and impulsivity” was excluded based on evidence suggesting its limited relevance to CPV (e.g., Rico et al., 2017). The BIS-11 is one of the most widely used self-administered impulsivity questionnaires, showing solid psychometric properties (reliability, validity, and factor structure), especially in young adult populations (Fossati et al., 2001; Stanford et al., 2009).

### 2.3. Procedure

The study was approved by the Ethics Committee of the University of Jaén, Spain (Ref. ABR.22/5.PRY). Participants were initially recruited through collaboration with university students. To expand the cohort, a snowball sampling technique was subsequently used. Specifically, the initial participants (first wave, *n* = 305, 29.3%), who were students from different ages, courses, and degree programs at the University of Jaén, recruited additional participants (second wave, *n* = 736, 70.7%) with limitations on the number and type of participants (50% university-50% non-university). This technique was used given the difficulty of directly contacting university students who continue to live with their parents, and especially non-university students, and to equalize the two types. All participants provided explicit informed consent after being informed about their rights and the objectives and procedure of the research. Participation was voluntary and anonymous, and each participant was assigned a unique identification code. Finally, data were collected via Google Forms, without financial incentives.

### 2.4. Data Analysis

Regarding data preprocessing, no missing data were recorded, as responses to the main study variables were mandatory in the online questionnaire. Data quality checks were conducted prior to analysis. Two exclusion criteria were applied to the initial sample to ensure the contextual validity of the responses and the consistency validity of the responses. Specifically, participants who reported no contact with their father or mother during adolescence and during the past year were excluded, as were those showing discrepancies of up to two points in their responses to two control items (items with equivalent content to the original item but slightly reformulated). This procedure allowed us to exclude both participants who lacked relational experiences related to the constructs analyzed and those who exhibited an inconsistent response pattern (*N* = 104). Additional analyses were conducted with the final sample (*N* = 1041) to detect outliers and influential cases. As detailed below, no evidence was found of observations that could compromise the stability of the results. Several reliability indices (Cronbach’s alpha, ordinal alpha, McDonald’s omega, and ordinal omega) were calculated, with the coefficients based on polychoric correlations (ordinal alpha and ordinal omega) being the most appropriate indicators of internal consistency for the instruments used in the present study, given the ordinal nature of the response scales (Dunn et al., 2014; Flora, 2020). Values of ≥0.70 are considered acceptable (Hair et al., 2019).

Concerning the statistical strategy, first, a Spearman correlational analysis was performed to examine the associations between the independent variables (punitive discipline by the father and the mother), the moderator (impulsivity), and the dependent variables (child-to-parent violence toward the father and the mother). Effect sizes for correlations were interpreted following conventional guidelines (Cohen, 1988, 1992), where values of 0.10, 0.30, and 0.50 indicating small, medium, and large effects, respectively.

Second, the assumptions of the regression analyses were examined. Multicollinearity was assessed using tolerance values and the variance inflation factor (VIF). All values were found to be within acceptable ranges, indicating the absence of multicollinearity among the predictors. The normality of the residuals was evaluated through histograms and Q-Q plots inspection, as well as with the Shapiro–Wilk test. The results showed statistically significant deviations from normality, although located mainly in the upper tail of the distribution. Homoscedasticity was examined through the inspection of scatterplots between standardized residuals and predicted values, revealing a mostly random distribution, without systematic funnel-like patterns, although with slightly greater dispersion of residuals at higher predicted values. These patterns in the residuals are consistent with the nature of the analyzed variables (violence) in the target sample (community population), characterized by a high concentration of low scores and a low concentration of high scores (e.g., Beckmann, 2020). Regarding the analysis of potential outliers, standardized residuals indicated that between 1.6% and 1.9% of the cases had values greater than ±3, which is within the expected ranges for large samples and does not indicate substantial violations of the model assumptions (Kline, 2015). In addition, the assessment of influential cases using Cook’s distance showed low values below the conventional critical threshold of 1, indicating that no individual cases exerted a disproportionate influence on the models estimates (Cook, 1977). Finally, Harman’s single-factor test was conducted to examine the potential influence of common method variance, showing that the first factor accounted for 20% of the total variance, which is below the commonly used threshold of 50%. This indicates that no single dominant factor emerged and suggests that common method bias is not a substantial issue in the data (Podsakoff et al., 2003).

Third, two simple moderation analyses were performed for each dependent variable to examine the conditional effects of impulsivity on the relationship between parental punitive discipline and CPV, controlling for sex and age. These analyses evaluated the effect that the predictor variable (parental punitive discipline) has on the dependent variables (CPV toward the father and the mother) at different levels of the moderating variable (impulsivity) to determine the extent to which high (+1 standard deviation above the mean) and low (−1 standard deviation below the mean) levels of the moderating variable can moderate the relationship between the dependent and independent variables. Additionally, in order to analyze the moderation pattern in more detail, the Johnson-Neyman technique was applied. The bias-corrected nonparametric bootstrap method was used to estimate the coefficients of the models, generating 5000 resamples and 95% bootstrap confidence intervals (Hayes, 2017). Direct and conditional effects were considered statistically significant when the confidence interval did not include zero. This approach provides more robust inferences and is less sensitive to possible deviations from the classical model assumptions (Alfons et al., 2022; Hayes, 2017; Preacher & Hayes, 2008). A sensitivity analysis was conducted using G*Power 3.1 (α = 0.05, 1 − β = 0.80), which indicated that, given the sample size and the number of predictors included in the moderation models, the study has sufficient statistical power to detect even small effect sizes, including interaction effects, which are typically more difficult to detect. Finally, when interactions were significant, simple slope plots were created to illustrate the moderating effect of impulsivity on the relationship between parental punitive discipline and CPV. Effect sizes were calculated using Cohen’s *f*^2^, where values of 0.02, 0.15, and 0.35 indicating small, medium, and large effects, respectively (Cohen, 1988).

## 3. Results

The correlational analysis showed positive and statistically significant associations among all study variables (see Table 1). In particular, violence toward fathers and mothers was positively related to parental punitive discipline by fathers and mothers. Specifically, the highest coefficients were found for the relationship between violence toward mothers and mother’s punitive discipline ρ (1041) = 0.505, *p* < 0.001, and between violence toward fathers and father’s punitive discipline ρ (1041) = 0.480, *p* < 0.001. Likewise, impulsivity was positively correlated with CPV toward both the father (ρ (1041) = 0.236, *p* < 0.001) and the mother (ρ (1041) = 0.315, *p* < 0.001), with a stronger correlation in the latter. The magnitude of the effect sizes ranged from moderate to high for the associations between CPV and parental punitive discipline, and from small to moderate for the associations between CPV and impulsivity.

The results of the moderation analyses (see Table 2) indicated that the models including paternal punitive discipline and impulsivity explained 23.2% of the variance in violence toward fathers (*R*^2^ = 0.232; *f*^2^ = 0.302) and 22.4% of the variance in violence toward mothers (*R*^2^ = 0.224; *f*^2^ = 0.288). In contrast, the models including maternal punitive discipline and impulsivity accounted for 17.1% of the variance in violence toward fathers (*R*^2^ = 0.171; *f*^2^ = 0.206) and 28.3% of the variance in violence toward mothers (*R*^2^ = 0.283; *f*^2^ = 0.394). These effect sizes ranged from moderate (*f*^2^ ≥ 0.15) to large (*f*^2^ ≥ 0.35), indicating that the models explained a meaningful proportion of variance in CPV, particularly in the case of maternal punitive discipline and impulsivity predicting violence toward mothers. From an applied perspective, the effect sizes suggest that parental punitive discipline and impulsivity may be relevant variables to consider in CPV prevention and intervention programs, also in young adult populations.

The inclusion of sex and age as covariates allowed for the estimation of the effects of parental discipline and impulsivity on CPV while controlling these sociodemographic variables. As shown in Table 2, these covariates were not significantly associated with CPV, with the exception of age in only one model. This suggests that the effects of parental punitive discipline and impulsivity remain robust after controlling for sex and age.

**Figure 1:**
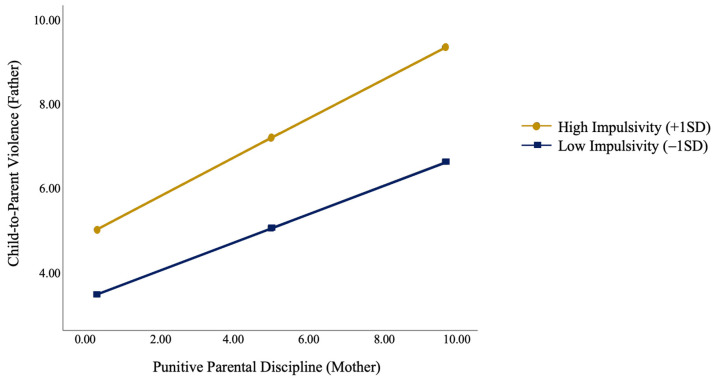
Interaction Effects of Impulsivity and Punitive Parental Discipline (Mother) on Child-to-Parent Violence (Father).

As shown in Table 2, for violence toward the father, model 1 (*F*(5, 1035) = 62.538, *p* < 0.001) revealed a significant direct effect of paternal punitive discipline (*B* = 0.600, *p* < 0.001) and impulsivity (*B* = 0.149, *p* < 0.001). However, the interaction term was not significant (*B* = −0.002, *p* = 0.677), suggesting no evidence of interaction effects in this model. This indicates that the relationship between punitive discipline by the father and violence toward the father does not vary according to levels of impulsivity. Model 2, which included maternal punitive discipline (*F*(5, 1035) = 42.772, *p* < 0.001), showed no significant direct effect on violence toward the father (*B* = 0.067, *p* = 0.718), although impulsivity remained a significant predictor (*B* = 0.112, *p* = 0.003). In this case, however, the interaction term was statistically significant (*B* = 0.009, *p* = 0.047). The results of the conditional effects are presented in Table 2 and illustrated in Figure 1. Specifically, the effect of maternal punitive discipline was significant at both low levels of impulsivity (−1*SD*) and high levels (+1*SD*). However, analysis of the moderation pattern using the Johnson-Neyman technique showed that the magnitude of the relationship between maternal punitive discipline and violence toward the father intensified as impulsivity increased. At the lower levels of impulsivity (score of 21.0), the effect of the maternal punitive discipline on violence toward the father was smaller (*B* = 0.266; 95% CI [0.090, 0.442]), whereas at higher levels (score of 68.0), the effect was substantially stronger (*B* = 0.712; 95% CI [0.420, 1.004]).

**Figure 2:**
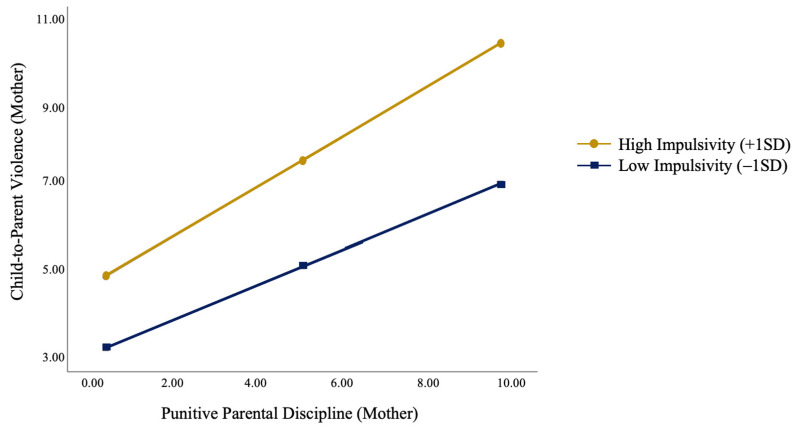
Interaction Effects of Impulsivity and Punitive Parental Discipline (Mother) on Child-to-Parent Violence (Mother).

Concerning violence toward the mother (see Table 2), model 1 (*F*(5, 1035) = 59.689, *p* < 0.001) showed no significant direct effect of paternal punitive discipline (*B* = 0.204, *p* = 0.225), a significant direct effect of impulsivity (*B* = 0.186, *p* < 0.001), and a non-significant interaction term (*B* = 0.006, *p* = 0.170), indicating no evidence of a moderating effect in this model. This suggests that the relationship between paternal punitive discipline and violence toward mothers does not vary as a function of impulsivity. In model 2, referring to maternal punitive discipline (*F*(5, 1035) = 81.724, *p* < 0.001), also showed no significant direct effect of this variable (*B* = 0.035, *p* = 0.834), while impulsivity remained a significant predictor (*B* = 0.124, *p* < 0.001), while showing a significant interaction term between discipline and impulsivity (*B* = 0.014, *p* = 0.002). Table 2 shows the results of the conditional effects, while Figure 2 illustrates these effects. In particular, the effect of the maternal punitive discipline was significant at both low levels of impulsivity (−1*SD*) and high levels (+1*SD*). Similar to the results of the model on violence toward the father, the Johnson-Neyman technique showed that the magnitude of the relationship between maternal punitive discipline and violence toward the mother intensified as impulsivity increased. Specifically, at the lower levels of impulsivity (score of 21.0), the effect of the maternal punitive discipline on violence toward the mother was of lesser magnitude (*B* = 0.320 (95% CI [0.159, 0.481]), while at the upper end of impulsivity (score of 68.0), the effect was considerably greater (*B* = 0.957 (95% CI [0.690, 1.225]).

## 4. Discussion

The present study examined, in a sample of Spanish young adults, the moderating role of impulsivity in the relationship between parental punitive discipline and CPV.

The first objective was to analyze the relationship between parental punitive discipline (father and mother) and CPV toward the fathers and mothers, as well as its predictive capacity. Hypothesis 1 predicted that both paternal and maternal punitive discipline would be positively associated with CPV toward both fathers and mothers and would be a significant predictor of both types of violence. In addition, a more intense effect is expected within the same than across parental figures. Numerous studies have documented the association between severe disciplinary styles and CPV (e.g., Beckmann et al., 2021; Cano-Lozano et al., 2022; Del Hoyo-Bilbao et al., 2018, 2020; Gámez-Guadix & Calvete, 2012; Pagani et al., 2009). The present findings indicate a positive association between paternal and maternal discipline and violence directed toward both fathers and mothers with moderate to large effect sizes. More specifically, the strongest correlations were observed between punitive discipline by the mother and violence toward the mother (0.505) and between punitive discipline by the father and violence toward the father (0.489). These findings are consistent with previous studies reporting similar sizes and stronger correlations within same-parent dyads (e.g., father–child and mother–child violence), with correlations ranging from 0.37 to 0.49 (Cano-Lozano et al., 2021b; Izaguirre & Calvete, 2017). These data confirm the strong relationship between punitive discipline and CPV and suggest certain reciprocity, such that the violence of young people toward their parents could represent responses to previous aggressions by the parents. On the other hand, in line with previous research (e.g., Del Hoyo-Bilbao et al., 2020; Jiménez-Granado et al., 2023), this study shows a strong correlation between CPV toward the father and CPV toward the mother. This finding suggests that the violence toward fathers and mothers should not be interpreted as completely independent phenomena, but rather as closely related manifestations of CPV.

Regarding its predictive value, only punitive discipline by the father had a significant direct effect on CPV toward the father, but not toward the mother. Punitive discipline by the mother did not have a direct effect on either violence toward the mother or toward the father. Therefore, the results, taken together, provide partial support for the proposed hypothesis. Previous findings regarding the roles of punitive discipline in CPV are inconsistent. Some studies identify corporal punishment and/or psychological aggression as predictors of CPV (Del Hoyo-Bilbao et al., 2018; Margolin & Baucom, 2014), both toward the father and toward the mother (Cano-Lozano et al., 2022; Pagani et al., 2009). However, in line with the present results, Jiménez-Granado et al. (2023) found that psychological aggression predicts CPV only toward fathers. It is important to note that many studies do not distinguish between paternal and maternal punitive discipline (e.g., Beckmann et al., 2021; Del Hoyo-Bilbao et al., 2018, 2020; Gámez-Guadix & Calvete, 2012; Pagani et al., 2009). This lack of differentiation may partly explain the distinct patterns of violence toward each parent observed in the present study. Future studies should also analyze the different types of violence separately. In addition, these mixed results suggest that the relationship between punitive discipline and CPV may depend on contextual or relational factors. In this sense, depending on which mediating or moderating factors are included in the model, the predictive role may vary and differ between father and mother. Likewise, the cited studies focus on childhood or adolescence, whereas the present study examines CPV during emerging adulthood, which may also account for these discrepancies.

The second objective was to examine the association between youth impulsivity and CPV, as well as its predictive value. Hypothesis 2 predicted that youth impulsivity would be positively associated with CPV toward both fathers and mothers and would be a significant predictor of both types of violence. The results fully confirmed the hypothesis. Impulsivity was positively associated with violence toward both the father and the mother. The magnitude of the correlations was similar to that found in samples of adolescents from the general population, with correlations of 0.24 (Calvete et al., 2011), although in our study, the correlations between impulsivity and CPV toward the mother were somewhat higher (0.315) than toward the father (0.236). Likewise, impulsivity was a direct predictor of violence toward both the father and the mother. These findings are consistent with prior research showing that impulsivity is associated with a higher probability of CPV in adolescents, both as a unidimensional construct (Calvete et al., 2011; Contreras & Cano-Lozano, 2015; Del Hoyo-Bilbao et al., 2020; Ibabe et al., 2007) and as a multidimensional construct (Rico et al., 2017). The results suggest that impulsivity is a key factor in CPV beyond adolescence.

The third objective was to explore the moderating role of youth impulsivity in the relationship between parental punitive discipline and CPV. Hypothesis 3 predicted that impulsivity would moderate this relationship, such that higher levels of impulsivity would strengthen the association between both paternal and maternal punitive discipline and CPV toward both fathers and mothers, with more intense effects within the same than between different parental figures. The hypothesis was partially supported, specifically only with regard to maternal punitive discipline. The results highlight impulsivity as a relevant individual factor in the association between maternal discipline and CPV, which becomes stronger as impulsivity increases. These findings are consistent with those reported by Gao et al. (2024), although their study found that the association between harsh parental discipline and CPV only emerged at high levels of impulsivity. Other research indicates that the association between inadequate parental discipline and CPV may be especially evident among adolescents with elevated levels of psychopathic or borderline personality traits (Jiménez-Granado et al., 2023). In the same way, the negative effects of punitive discipline are greater in the presence of stress, parental ineffectiveness, and impulsivity from parents (Cano-Lozano et al., 2022). This pattern is consistent with the diathesis-stress model (Monroe & Simons, 1991), which posits that individual vulnerabilities can intensify the impact of stressful experiences on maladaptive outcomes. In this context, impulsivity may function as a dispositional vulnerability that exacerbates the effects of parental harsh discipline on adolescents’ violent behavior and moral disengagement. Individuals with higher impulsivity tend to experience greater situational stress and less emotional regulation capacity (Y. Chen et al., 2021), increasing the likelihood of reacting to coercive parenting practices with anger and poorly controlled responses (Zhu et al., 2016). Consequently, they may be particularly sensitive to harsh disciplinary practices and more prone to respond with aggressive behavior during interactions with their parents. Nevertheless, alternative explanations should be considered, such as the possibility that impulsive youth led to harsher disciplinary responses from parents.

In contrast, impulsivity did not moderate the association between paternal punitive discipline and CPV toward either parent. This finding suggests that the relationship between paternal punitive discipline and CPV, in the present sample, is independent of the youth’s impulsivity. One possible explanation for the lack of significant moderation effects for paternal punitive discipline could be that such discipline is more normative and therefore operates in a more direct manner, and is less influenced by the children’s individual characteristics. Another possible explanation would be the differences in gender socialization within parent–child relationships. In certain contexts, paternal discipline is generally associated with more formal authority, less involvement in daily management, and more sporadic disciplinary intervention. In contrast, mothers are usually the primary caregivers, which increases the frequency of mother–child interactions and opportunities for conflict escalation, leading to more frequent disciplinary practices (Ulman & Straus, 2003). Studies using samples of Spanish adolescents have shown that both boys and girls rate their mothers higher on most dimensions of parenting style (Oliva et al., 2007). This finding aligns with studies conducted in other countries that consider mothers to be more involved, caring, and controlling than fathers (Laible & Carlo, 2004). Taken together, these data are consistent with several international studies that confirm that CPV is directed more toward the mother (Burgos-Benavides et al., 2025; Contreras et al., 2026; Navas-Martínez et al., 2025). However, these aspects mentioned were not evaluated in the present study. Future studies should specifically examine the role of exposure time in parent–child relationships and the frequency of disciplinary practices in the development of CPV.

Furthermore, punitive discipline may also be interpreted differently depending on the parent. Maternal warmth could be expected, so punitive behavior from mothers may be interpreted as rejection or may provoke a more intense emotional response, amplified by a lack of self-regulation strategies. On the other hand, the reaction to paternal punitive discipline may depend less on emotional control and be more related to other variables, such as power struggles, like dominance–submission, or the imitation of gender roles. The fact that paternal punitive discipline has a more direct effect on CPV toward the father could be because it operates at the level of authority, functioning as a model of coercive behavior perceived as an exercise of power. It would not be processed at an emotional level. In this sense, impulsivity (emotional regulation) would not play such a significant role; it would be simply the application of a dominance strategy learned at home. In this sense, it is of interest to specifically analyze in future studies the expectations and reactions regarding the discipline of the father and the mother, and their role in the development of violence towards both the father and the mother.

In summary, the fact that impulsivity moderates the relationship between maternal punitive discipline and CPV may be due to it occurring in a more frequent and emotional relational context. Overall, these findings underscore the importance of considering both the individual characteristics of young people and the specific relational dynamics with their parents when examining the process involved in the development of CPV.

Importantly, the moderating role of impulsivity differed depending on the target of the violence. The interaction between maternal punitive discipline and impulsivity was stronger when CPV was directed toward mothers than toward fathers. Even at lower levels of impulsivity, maternal punitive discipline was more strongly associated with violence toward mothers than toward fathers, and this difference became more pronounced at higher levels of impulsivity. Furthermore, the empirical literature on CPV often shows different patterns of violence toward fathers and mothers, indicating that some factors may be more involved in violence directed at fathers and others in violence toward mothers, or that both may operate in different ways (Calvete et al., 2015). Further research is needed to clarify the role of sex in the link between parenting practices and CPV.

In terms of explained variance, paternal punitive discipline and impulsivity accounted for 23.2% of the variance in violence toward the father and 22.4% toward the mother, whereas maternal punitive discipline and impulsivity explained 28.3% toward the mother and only 17.1% of the variance toward the father. Even the effects of these variables remain robust when controlling for sex and age (with the exception of age in only one model). These coefficients of determination represent moderate to large effects on the explained variance, suggesting that a substantial proportion -approximately one-fifth to more than one-quarter- of CPV is associated with the combination of punitive discipline and impulsivity. In practical terms, this implies that interventions aimed at reducing punitive discipline practices and improving youth’s self-control could significantly decrease levels of CPV. The stronger association observed for maternal punitive discipline in relation to violence toward mothers may reflect greater exposure or relational proximity, highlighting the importance of tailoring family-based interventions according to parent–child dynamics. In addition, given that CPV is a complex, multicausal phenomenon, this suggests that, while these data are significant, other variables not included in the model may be influencing the results (e.g., anger, moral disengagement, peer influence, mental health, or substance use).

Several limitations should be considered when interpreting the results of this study. First, the cross-sectional design does not allow for establishing causal relationships. Longitudinal studies are needed to clarify the temporal relationship between punitive discipline, impulsivity, and CPV.

Second, in the present study, punitive discipline was assessed by asking young adults about their parents’ behavior during adolescence (between the ages of 12 and 17). Although this period is relatively close to the present (ages 18 and 25), it may introduce recall bias. Furthermore, the questions refer to specific behaviors, and the response scale is qualitative frequency, which allows reporting of habitual behavior patterns and thus reduces recall bias. Previous research suggests that retrospective reports of childhood experiences can provide reasonably valid information (Hardt & Rutter, 2004), particularly when referring to frequent and stable experiences, such as parenting practices (Schwarz, 1999). In the case of CPV and punitive discipline, it would also be interesting to analyze the different forms of violence. This could provide information on a possible correspondence between the type of violence suffered and that perpetrated, as well as identify possible differentiating mechanisms depending on the type of violence.

Third, all variables were assessed through self-report by a single informant, which could introduce common method variance. Although Harman’s test does not suggest the presence of a dominant factor in the data, this procedure does not allow for the complete exclusion of the possible influence of common method variance. To overcome this limitation, future studies should employ multiple informants and assessment methods.

Finally, several aspects of the sampling procedure and geographical context limit the generalizability of the findings. The study was based on a non-probability sample (snowball technique), which introduces selection bias that may, among other aspects, underrepresent the most severe cases of CPV. The data are from a Spanish context, so the specific characteristics of this context must be taken into account. In Spain, late emancipation often forces young people between the ages of 18 and 25 to continue living with their parents, increasing the risk of everyday conflicts. This differs from Anglosphere contexts, where early independence can mitigate such conflicts. Likewise, the Spanish socialization context, unlike others, is characterized by the primacy of the indulgent parenting style, which combines high warmth with low coercion (García & Gracia, 2010). This implies that the results are not generalizable to other sociocultural contexts. Future studies should aim to replicate the results in other contexts and diverse samples.

Despite these limitations, this study contributes to the understanding of CPV by confirming the role of parental punitive discipline and impulsivity in CPV and, more importantly, analyzing the interaction between family factors (punitive discipline) and individual factors (impulsivity). Furthermore, it is conducted on a sample of young, non-emancipated adults, a population that has received little attention in the scientific literature on the subject to date, which has focused primarily on adolescents. Importantly, the study distinguishes between paternal and maternal punitive discipline as well as between CPV directed toward both father and mother, providing a more nuanced understanding of these dynamics.

In line with the models that have served as the theoretical framework for this study, the findings suggest that punitive discipline may function as a form of modeling, whereby individuals internalize the use of violence as a means of resolving conflict, thus reproducing this behavior in their parents. Furthermore, there are individual vulnerability factors, such as impulsivity, that may intensify the relationship between punitive discipline and CPV. However, the present findings suggest that the mechanisms involved in these relationships may vary depending on whether the discipline is exercised by the father or the mother. Further studies will need to delve deeper into this aspect.

Future research should also examine additional moderating and mediating factors, both individual and from the family and social contexts, to better understand the complex dynamics underlying CPV. In addition, it is necessary to deepen in sex differences in both children and parents. It would also be valuable to explore the differential influence of these factors on the different types of CPV (physical, psychological, and financial violence or control and domain behaviors toward parents) and different forms of parental punitive discipline (physical or psychological) because each type can partially reflect distinct processes.

The magnitude of the effects found in this study reinforces the practical relevance of the findings, especially given the complex and multifactorial nature of CPV. The proportion of variance explained suggests that punitive discipline and impulsivity are relevant variables for the CPV. In this regard, these findings have important practical implications for the prevention and intervention of CPV. Strategies should include a comprehensive approach that combines both family and individual levels interventions. At the family level, efforts should focus on eliminating punitive discipline that involves the use of physical punishment and psychological aggression, while promoting positive parenting strategies. At the individual level, interventions should aim to improve emotional regulation and impulse control in young perpetrators, thereby reducing the likelihood of violent interactions. The findings also suggest that CPV may operate through different mechanisms depending on who is exercising discipline. It is necessary to go beyond generic intervention approaches and consider modifications based on paternal versus maternal disciplinary patterns and their specific needs. In the case of fathers, intervention could focus on eliminating coercive models that reinforce the child’s aggressive behavior. In the case of mothers, it will be necessary to train them in non-reactive supervisory skills, enabling them to act as external regulators and adapt discipline to avoid triggering aggressive behavior. Therefore, intervention programs should involve both fathers and mothers and base the interventions on the differential needs in the parenting practices of each, and in relation to the individual characteristics of the children. Integrating these approaches may enhance the effectiveness of prevention and intervention programs.

## Figures and Tables

**Table 1:** Correlations Among Study Variables.

	1	2	3	4	5
1. Child-to-Parent Violence (Father)	-				
2. Child-to-Parent Violence (Mother)	0.832	-			
3. Parental Punitive Discipline (Father)	0.480	0.430	-		
4. Parental Punitive Discipline (Mother)	0.403	0.505	0.712	-	
5. Impulsivity	0.236	0.315	0.229	0.274	-

Note. *N* = 1041. All correlations are significant at *p* < 0.001.

**Table 2:** Moderating Role of Impulsivity in The Relationship Between Parental Punitive Discipline and Child-to-Parent Violence.

		Child-to-Parent Violence (Father)			Child-to-Parent Violence (Mother)		
Model		*B*	*t*	95% CI	*B*	*t*	95% CI
1	Direct and interaction effects						
	Punitive Discipline (Father)	0.600	3.539 ***	[0.267, 0.932]	0.204	1.215	[−0.126, 0.534]
	Impulsivity	0.149	4.214 ***	[0.080, 0.219]	0.186	5.292 ***	[0.117, 0.255]
	Punitive Discipline (Father) * Impulsivity	−0.002	−0.417	[−0.010, 0.007]	0.006	1.375	[−0.002, 0.014]
	Sex	0.208	0.609	[−0.463, 0.880]	0.377	1.111	[−0.289, 1.043]
	Age	−0.149	−1.695	[−0.322, 0.024]	−0.194	−2.228 *	[−0.365, −0.023]
2	Direct and interaction effects						
	Punitive Discipline (Mother)	0.067	0.361	[−0.296, 0.429]	0.035	0.209	[−0.297, 0.368]
	Impulsivity	0.112	2.985 **	[0.038, 0.186]	0.124	3.586 ***	[0.056, 0.191]
	Punitive Discipline (Mother) * Impulsivity	0.009	1.991 *	[0.001, 0.019]	0.014	3.104 **	[0.005, 0.022]
	Sex	−0.030	−0.084	[−0.727, 0.667]	0.216	0.663	[−0.423, 0.855]
	Age	−0.097	−1.063	[−0.277, 0.082]	−0.141	−1.688	[−0.306, 0.023]
	Conditional effects						
	−1*SD* below the mean of Impulsivity (28.8)	0.340	5.824 ***	[0.226, 0.455]	0.426	7.961 ***	[0.321, 0.532]
	+1*SD* above the mean of Impulsivity (42.3)	0.468	10,501 ***	[0.380, 0.555]	0.608	14.904 ***	[0.528, 0.689]

Note. *N* = 1041. * *p* < 0.05, ** *p* < 0.01, *** *p* < 0.001.

## Data Availability

The raw data supporting the conclusions of this article will be made available by the authors, without undue reservation.
